# 1607. Clinical Outcomes at Month 6 after Initiation of Cabotegravir and Rilpivirine Long-Acting (CAB + RPV LA) in an Observational Real-World Study (BEYOND)

**DOI:** 10.1093/ofid/ofad500.1442

**Published:** 2023-11-27

**Authors:** Gary I Sinclair, Michael Sension, Alexandra Dretler, Stefan Schneider, Catherine K Schubert, Deanna Merrill, David Richardson, Bintu Sherif, Laurie Zografos, Cindy Garris

**Affiliations:** PrismHealth NTX, Dallas, Texas; CAN Community Health, Fort Lauderdale, Florida; Infectious Disease Specialists of Atlanta, Decatur, Georgia; Long Beach Education and Research Consultants, Long Beach, CA, USA, Long Beach, California; ViiV Healthcare, RTP, North Carolina; ViiV Healthcare, RTP, North Carolina; RTI Health Solutions, Research Triangle Park, North Carolina; RTI Health Solutions, Research Triangle Park, North Carolina; RTI Health Solutions, Research Triangle Park, North Carolina; ViiV Healthcare, RTP, North Carolina

## Abstract

**Background:**

CAB+RPV LA is the first complete long-acting regimen for virologically suppressed people with HIV (PWH) and demonstrated non-inferiority to standard of care antiretroviral regimens in the Phase 3/3b trials FLAIR, ATLAS, ATLAS-2M, and SOLAR. Implementation of a provider administered regimen poses new delivery challenges and real-world evidence is essential to understand utilization and clinical outcomes. The BEYOND study describes the demographics and month 6 (M6) clinical outcomes of patients initiating CAB+RPV LA in the US.

**Methods:**

BEYOND is a 2-year observational real-world study of utilization, outcomes, and experience of people with HIV (PWH) initiating CAB+RPV LA (monthly or every 2 months) across 30 US sites. Healthcare providers (HCPs) completed an electronic case report form (eCRF) at baseline and M6 to capture demographics, medical and treatment history, and clinical outcomes.

**Results:**

A total of 308 PWH (Table 1) were enrolled between Sep 2021- Jul 2022 and initiated on CAB+RPV LA. As of the data cut-off for this analysis (Jan 2023), 248 PWH had reached M6 of which 25 were reported as having discontinued CAB+RPV LA. The most common HCP reported primary reason for initiating CAB+RPV LA was patient request (41%). At M6, of the 803 injections given after the first injections, 667 (83%) occurred within +/-7 days of the target treatment date and 136 (17%) were outside the target treatment window (median 4 days outside). Of 1087 total injections expected, 44 (4%) were missed; of these, 3 (7%) used oral CAB+RPV and 19 (44%) used other oral regimens to cover missed injections. Of 189 PWH with viral load data available at both baseline and M6 (Table 2), 181 (96%) had viral loads of < 50 copies/mL. Confirmed virologic failure (CVF) occurred in 4 (1.6%), including 1 who had missed injections. Resistance was reported in 2 PWH. Discontinuations due to drug intolerance/injection site reactions were reported in 6 PWH.

**Figure:**
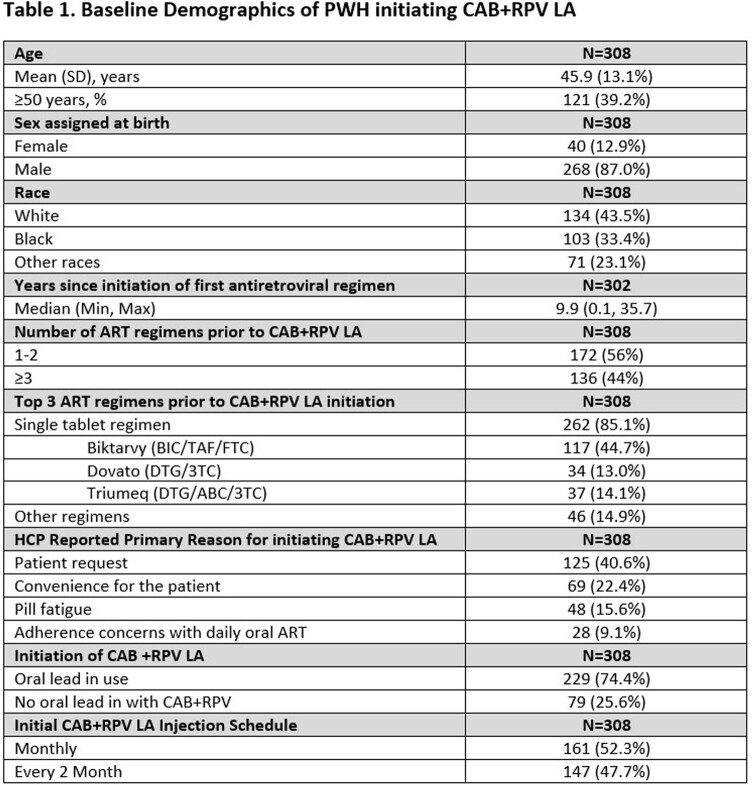

**Figure:**
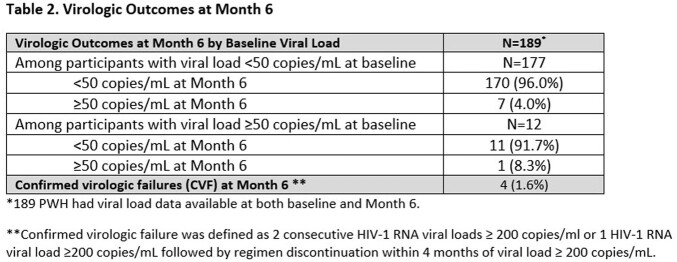

**Conclusion:**

The M6 results from real world initiation of CAB+RPV LA in the US are consistent with the Phase 3/3b clinical trials with high rates of virologic suppression, low rates of CVFs and treatment emergent resistance, and low rates of discontinuation due to drug intolerance.

**Disclosures:**

**Gary I. Sinclair, MD**, Abbvie: Grant/Research Support|Gilead: Advisor/Consultant|Gilead: Grant/Research Support|Janssen: Advisor/Consultant|Janssen: Grant/Research Support|Janssen: Honoraria|Merck: Advisor/Consultant|Merck: Grant/Research Support|Merck: Honoraria|Thera: Advisor/Consultant|Thera: Grant/Research Support|Thera: Honoraria|ViiV: Advisor/Consultant|ViiV: Grant/Research Support|ViiV: Honoraria **Michael Sension, MD**, Gilead: Advisor/Consultant|Gilead: Honoraria|Viiv: Advisor/Consultant|Viiv: Grant/Research Support|Viiv: Honoraria **Alexandra Dretler, MD**, Gilead: Stocks/Bonds|Johnson and Johnson: Stocks/Bonds|Pfizer: Stocks/Bonds **Stefan Schneider, MD**, ViiV Healthcare: Grant/Research Support **Catherine K. Schubert, PharmD**, GSK: Stocks/Bonds|ViiV Healthcare: Employee of ViiV Healthcare **Deanna Merrill, PharmD, MBA, AAHIVP**, ViiV Healthcare: Employment|ViiV Healthcare: Stocks/Bonds **Cindy Garris, MS**, GSK: Stocks/Bonds|ViiV Healthcare: Employee

